# Visual Behaviour During the Interpretation of Cone-Beam Computed Tomography Images of Traumatic Dental Injuries: An Eye-Tracking Study

**DOI:** 10.7759/cureus.68202

**Published:** 2024-08-30

**Authors:** Shatha S Zahran, Maha S Alghamdi, Maryam M Babutain, Hanadi M Khalifa

**Affiliations:** 1 Endodontics, Faculty of Dentistry, King Abdulaziz University, Jeddah, SAU; 2 General Dentistry, Faculty of Dentistry, King Abdulaziz University, Jeddah, SAU; 3 Oral Diagnostic Science Department/ Oral Radiology, Faculty of Dentistry, King Abdulaziz Univeristy, Jeddah, SAU

**Keywords:** cone-beam computed tomography (cbct), interpretation, traumatic dental injuries, eye tracking, visual behaviour

## Abstract

Background: This cross-sectional observational study aimed to investigate the visual attention patterns of postgraduate endodontic residents during the interpretation of cone-beam computed tomography (CBCT) scans for traumatic dental injuries (TDIs) using eye-tracking technology.

Methods: A cohort of 10 residents who were recruited from King Abdulaziz University Dental Hospital (KAUDH) underwent interpretation of seven CBCT images of TDIs. Eye-tracking metrics, including dwell time, entry time, end time, and the number of revisits, were recorded and analyzed using nonparametric statistical tests.

Results: Eye-tracking data revealed that patients with lateral luxation and extrusive luxation pathologies had the longest mean dwell times (1.82 seconds and 1.50 seconds, respectively). These findings were statistically significant compared to other pathologies (p<0.001). Conversely, horizontal root fractures and periapical radiolucency were identified more quickly by the participants (mean entry times of 6.60 seconds and 8.84 seconds, respectively).

Conclusions: The findings indicate variability in visual attention metrics depending on the type of TDI, suggesting that certain injuries may require more focused attention for accurate diagnosis. Specifically, lateral and extrusive luxation injuries attracted longer dwell times, possibly due to their unique diagnostic challenges. This research provides a basis for future studies aiming to optimize education and training related to CBCT interpretation of traumatic dental injuries.

## Introduction

Traumatic dental injuries (TDIs) are acute injuries that affect teeth, periodontium, and surrounding soft tissue due to trauma, necessitating immediate clinical intervention. Such injuries can induce instantaneous deleterious effects on dental structures and are precursors to an array of subsequent pathological outcomes, including but not limited to pulp necrosis, inflammatory resorption, marginal bone loss, internal root resorption, and ankylosis [1, 2]. The types of TDI can be classified into enamel infraction, enamel fracture, enamel-dentine fracture, complicated crown fracture, crown-root fracture, root fracture, and various forms of luxation injuries, such as concussion, subluxation, extrusive luxation, lateral luxation, intrusive luxation, and avulsion [3]. Studies have highlighted that maxillary central incisors are most frequently affected, with complicated crown fractures constituting between 2% and 13% of all dental injuries [4].

Comprehensive and accurate clinical and radiographic examinations are essential for determining the appropriate management to follow. Clinical examinations must include percussion tests and sound tests, palpation tests, mobility tests, and pulp vitality/sensibility tests. Radiographic assessment is a crucial aspect of the initial examination [5]. In most cases, an intraoral two-dimensional periapical (PA) radiograph is the first choice for evaluating traumatic teeth, although its accuracy is suboptimal, especially in cases of root fracture or alveolar fracture [6]. The advent of three-dimensional imaging via cone-beam computed tomography (CBCT) has revolutionized the diagnosis and treatment planning of TDIs in endodontics [7]. The CBCT technology offers an enhanced level of diagnostic accuracy compared to traditional two-dimensional radiographic techniques, thereby instilling greater confidence in the clinical decision-making process for TDIs [8]. According to the American Association of Endodontists, limited field-of-view CBCT scans are the preferred imaging modality for the assessment and management of localized dentoalveolar trauma [9]. A CBCT allows for better visibility of TDIs, especially root fractures, crown/root fractures, and lateral luxation. The location, extent, and direction of a fracture can be determined via CBCT [10].

Originally invented in the late 18^th^ century by Edmund B. Huey, eye-tracking technology constitutes a sophisticated means of capturing ocular behavior by identifying the human pupil and recording eye movements and fixations during the visual inspection of images or web content [11]. This technology facilitates the quantification of specific oculomotor parameters, including gaze, fixation, saccades, and overall eye movement trackability [12]. The term "gaze" denotes the focal point of an individual's visual attention on an image, while "fixation" refers to the point at which their gaze stops moving to take information. The duration of fixation can vary significantly, ranging from 100 to 500 milliseconds, contingent upon the nature of the visual stimuli; however, while engaged in reading activities, fixation averages approximately 250 milliseconds [12].

Numerous studies have confirmed the value of eye-tracking technologies in the medical field [13-17]. In dentistry, numerous investigations have been undertaken to assess the utility of periapical and panoramic radiographs, often employing eye-tracking technology as a methodological tool [18-24]. In endodontics, eye-tracking technology has been employed in a pilot study to evaluate fixation and scan patterns among observers of varying experience levels using periapical radiographs. The main observation was that areas of high contrast in periapical radiographs garnered more attention than did standard regions [22]. The use of eye tracking for scanning CBCT has been reported in only one pilot study aiming to document the eye movements of 20 endodontists interpreting CBCT images of maxillary posterior and mandibular anterior teeth [25]. In this study, three CBCT interpretation strategies were identified: "non-systematic/nontargeted," "scanning," and "drilling." This study revealed that a systematic approach to CBCT analysis resulted in extended fixation durations and enhanced detection of pathological conditions compared to non-systematic methods.

To date, no published studies have explored the visual interpretation of CBCT in TDIs. Therefore, the objective of this observational cross-sectional study was to analyze the visual fixation and other visual metrics of endodontic residents viewing CBCT images of various TDIs in the anterior maxilla using eye-tracking technology. The objective of this study was to establish whether the complexity or type of TDI influences the diagnostic observation process of endodontic residents by comparing and measuring these visual metrics. The findings of this study may provide insight into the perceptual challenges that dentists face when using CBCT imaging to diagnose various TDIs and may contribute to the creation of more efficient training approaches in endodontic education and practice. This study has the potential to enhance diagnostic accuracy, leading to better patient treatment outcomes. The null hypothesis tested in this study was that there are no variations in the visual metrics of CBCT images depicting different TDIs, as observed using eye-tracking technology.

## Materials and methods

This study was approved by the Institutional Research Board and the Research Ethics Committee at King Abdulaziz University Dental Hospital (KAUDH) with the ethical clearance number 4449727. ​​

Subjects

Participants were endodontic residents (regardless of the level of training) currently enrolled in a clinical endodontic postgraduate program at KAUDH. All participants received mandatory training in CBCT interpretation as part of the postgraduate curriculum. This training was conducted in collaboration with the oral and maxillofacial radiology departments and covered both theoretical and practical aspects. The curriculum included lectures on the principles of CBCT technology, image acquisition, and common artifacts, as well as hands-on sessions where residents were guided through the analysis of a wide range of CBCT scans. Emphasis was placed on identifying anatomical landmarks, diagnosing pathology, and understanding the limitations of CBCT in clinical practice. Additionally, residents were required to complete a series of case-based assessments to ensure proficiency in CBCT interpretation before being involved in this study. Initially, a total of 13 postgraduate endodontic residents in the first, second, and third years were invited via email to briefly explain the aim of the study. Participants who had strabismus of more than one prism diopter in both eyes were excluded. Therefore, after exclusion due to strabismus, 10 residents participated in this study. The observers were free to withdraw from the study at any time, and no incentives were provided. 

Experimental material

Seven CBCT examinations of patients with various TDIs, including crown fracture, root fracture, alveolar bone fracture, and luxation injuries, were selected by a Delphi panel consisting of an endodontist and an oral and maxillofacial radiologist from the oral and maxillofacial radiology department at KAUDH. Patients who visited the endodontic department with a history of trauma to the maxillary anterior teeth between January 2018 and December 2022 were selected. All patient identifiers were anonymized, and serial numbers were given to maintain confidentiality.

**Figure 1:**
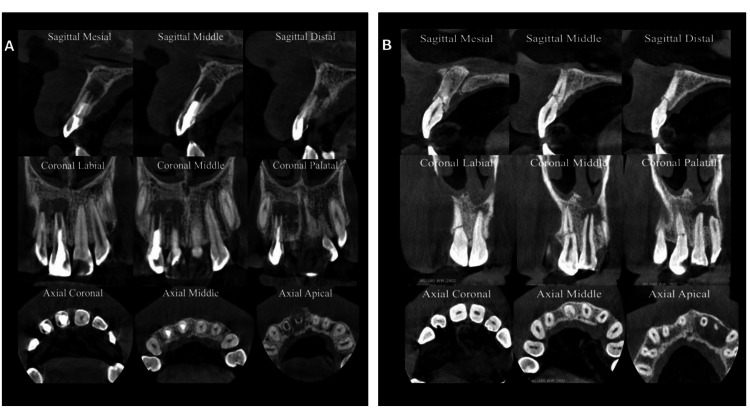
Illustrative CBCT images presented to participants in the study (A) A case of an uncomplicated crown fracture, accompanied by periapical radiolucency, identified in tooth #12; (B) A case of horizontal root fracture observed in tooth #11 CBCT: cone-beam computed tomography

The CBCT images of the anterior maxillary region were acquired using a KaVo 3D Pro machine (Instrumentarium Dental, PaloDEx Group Oy, Nahkekelantie, Finland). The CBCT acquisition parameters were 89 kilovoltage peak (kVp), 6 milliamperes (mA), 5 x 5 cm field of view, and 0.085 mm voxel size (endo-resolution mode). For each CBCT case, nine images (three axial, three coronal, and three sagittal images at different levels) were extracted using OnDemand 3D Imaging Software (Cybermed, Seoul, South Korea), as shown in Figure 1. The participants were shown periapical images and CBCT images arranged in a PowerPoint presentation (Microsoft Corp., Redmond, WA) for the seven experimental cases. Before commencing the experiment, a preliminary investigation was conducted to check the arrangement of the slides and the quality of the images in terms of resolution, brightness, and contrast.

Eye tracking and experimental setup

A PowerPoint presentation was presented using Experiment Center software (Sensomotoric Instruments, Teltow, Germany) for the seven TDI cases. At the beginning of each case, a detailed history and clinical findings were provided. This was followed by a periapical image and then nine CBCT images. The images were displayed on a 15.6-inch laptop with a 1600*900 resolution. The data were collected using a RED-m® SMI eye tracking device (Sensomotoric Instruments) that was attached to the lower edge of the laptop by a magnetic strip, which allows comfortable head positioning.

**Figure 2:**
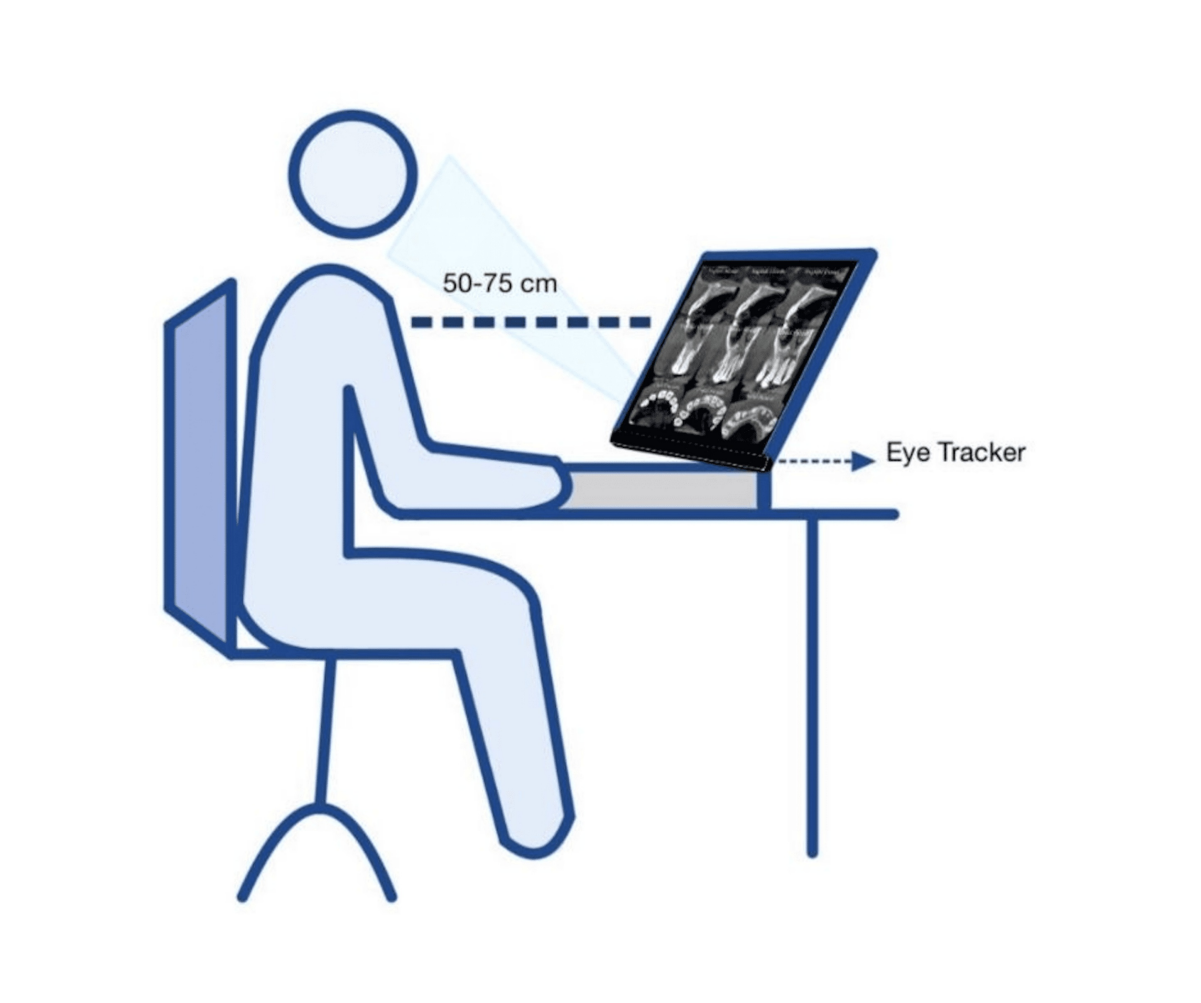
Eye tracking experimental setup This image is an original creation by the authors. It was created using Clip Studio Paint, version 3.1.2 (Celsys, Inc., Tokyo, Japan)

Detailed written instructions were given to all participants, followed by the consent form. Each participant was invited to sit in a dimly lit, quiet room facing the wall with a laptop computer screen placed in front of them to minimize distractions. The operating distance between the device and the observer's eyes was between 50 cm and 75 cm. Participants were seated on a stable, not height-adjustable chair to prevent unwanted movements and were positioned in the center of the camera's field of view. Finally, a calibration procedure was carried out utilizing a five-point calibration image to guarantee accurate alignment of the gaze pattern with respect to the image. The precision (gaze deviation) was set at 0.5°, which translates to a 5 mm spatial resolution at a 65 cm distance from the eye to the screen as shown in Figure 2.

Data collection and analysis

Eye-tracking outputs were simultaneously collected using Experiment Centre software (Sensomotoric Instruments). Data analysis was carried out using BeGaze (Sensomotoric Instruments), and the data were exported into Microsoft Excel, version 16.66.1 (Microsoft Corp.). The following metrics were extracted from the eye-tracking software and recorded for analysis: number of revisits, entry time, dwell time, and end time, as shown in Table 1.

**Table 1:** List of the visual metrics used for the area of interest (AOI), their definitions and units of measurement

Visual metrics	Unit	Description
Entry time	Seconds	Mean time from the start of the image examination to the first identification of the AOI
End time	Seconds	The total amount of time spent on one image
Dwell time	Seconds	The amount of time spent looking within an AOI
Revisits	Count	The number of revisits to the AOI once the observer leaves the AOI the first time

The time duration data were converted into seconds. All analyses were carried out using IBM SPSS Statistics software for Windows, version 28 (IBM Corp., Armonk, NY). The data from all participants for each patient were pooled for the analysis. Descriptive statistics were used to summarize various study characteristics, including overall mean dwell time, entry time, end time, and number of revisits for each case. Cases were grouped based on different types of trauma/pathology detected on CBCT as follows: external resorption, lateral luxation, cortical bone fracture, extrusive luxation, periapical radiolucency, horizontal root fracture, and uncomplicated crown fracture. The data were tested for normality with the Kolmogorov-Smirnov test and the Shapiro-Wilk test and were found to be nonparametric. Therefore, the Kruskal-Wallis test was used to compare the dwell time, entry time, end time, and number of revisits among patients with different TDIs detected via CBCT.

## Results

The seven CBCT scans in this study had a total of 32 areas of interest (AOIs). Horizontal root fracture was the type of trauma associated with the greatest number of AOIs, while lateral luxation and cortical bone fracture were the least common. These data are presented in Table 2.

**Table 2:** Number of areas of interest (AOI) in the selected cases All AOIs were recognized by endodontic residents and recorded using an eye-tracking device.

AOI	N
Horizontal/oblique root fracture	14
periapical radiolucency	8
Extrusive luxation	2
External resorption	2
Lateral luxation	1
Cortical bone fracture	1
Non-complicated crown fracture	4
Total	32

All AOIs related to all TDIs were identified and recognized by residents using the eye-tracking device. However, the visual parameters varied between different types of TDIs. Patients presented with lateral and extrusive luxation had mean dwell times of 1.82 seconds and 1.50 seconds, respectively, which were greater than those with other types of TDIs (Figures 1, 2). The differences in dwell time, entry time, and end time between different types of TDIs were significant (Kruskal-Wallis test, p<0.001), as shown in Table 2. The number of revisits was also greater in patients with lateral and extrusive luxation, but the difference did not reach statistical significance (p = 0.251). Participants identified horizontal root fracture and periapical radiolucency faster (mean entry times of 6.60 seconds and 8.84 seconds, respectively) than external resorption and uncomplicated crown fracture (mean entry times of 18.8 seconds and 20.82 seconds, respectively). 

**Figure 3:**
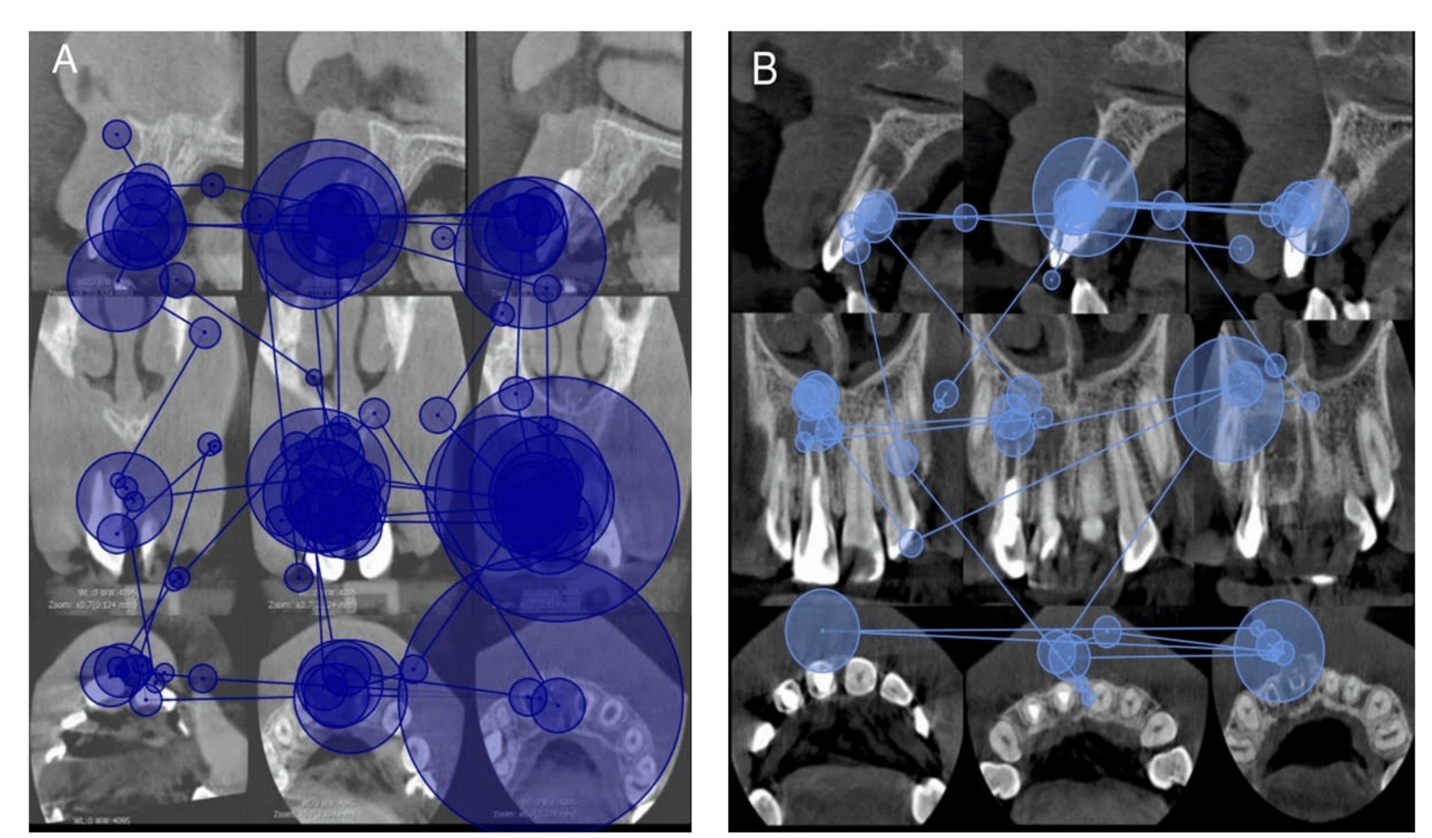
Coronal, sagittal, and axial cone-beam computed tomography images, highlighting (A) tooth #11 with lateral luxation and (B) tooth #12 exhibiting periapical radiolucency. The diagram features varying sizes of blue circles, which signify the participants' mean gaze duration (dwell time) on the specified regions of interest. The larger circles associated with lateral luxation in (A) than in (B) are shown.

**Figure 4:**
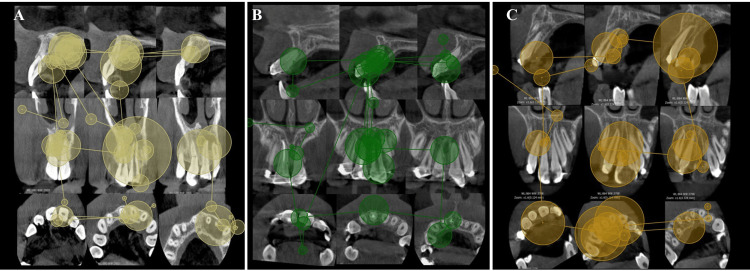
Eye-tracking results for various TDI cases of a single participant This figure illustrates various cases analyzed in the study, each accompanied by eye-tracking data for an individual participant. Fixations are represented by circles, with larger circles representing longer fixations and smaller circles representing shorter fixations. (A) A case of horizontal root fracture in tooth #12. The mean entry time for this image was 6.60 seconds, showing rapid identification of the area of interest. (B) A case of extrusive luxation in tooth #21. The mean end time for this image was 39.37 seconds, indicating that participants spent a longer duration observing this scan than observing the horizontal root fracture scan (B). (C) A case of cortical bone fracture in the palatal region. The mean dwell time for this image was 0.56 seconds, while the mean end time was 42.35 seconds. These metrics indicate that despite the short time spent looking at the AOI, participants allocated the most time to scan the entire CBCT image in comparison to other pathological conditions. TDI: traumatic dental injuries; CBCT: cone-beam computed tomography; AOI: area of interest

The difference in dwell time, entry time, and end time between different types of pathologies was significant. Dwell time in cases of lateral luxation and extrusive luxation was 1.82 seconds and 1.50 seconds, respectively, which was significantly higher than other types of trauma (p<0.001). Revisits were greater in cases with lateral and extrusive luxation but did not reach statistical significance (p = 0.251); all the visual metrics associated with each type of TDI were represented in Table 3. Cases were analyzed in this study, each accompanied by eye-tracking data (entry time, end time, and dwell time) for an individual participant. Fixations are represented by circles, with larger circles indicating longer fixations (Figures 3, 4).

**Table 3:** The means of the visual metrics of different TDIs Kruskal‒Wallis test; TDI: traumatic dental injury

Type of TDIs	Entry time (seconds)	End time (seconds)	Dwell time (seconds)	Revisits (count)
Overall mean	29.91 (± 0.73)	28.05 (± 1.33)	01.06 (± 0.08)	0.88 (± 0.1)
External resorption	18.81 (± 3.16)	36.91 (± 2.62)	0.65 (± 0.16)	0.5 (± 0.2)
Lateral luxation	14.38 (± 2.99)	34.03 (± 3.86)	1.82 (± 0.48)	1.9 (± 0.6)
Cortical bone fracture	14.19 (± 4.65)	42.35 (± 6.45)	0.56 (± 0.16)	0.3 (± 0.3)
Extrusive luxation	13.92 (± 2.21)	39.37 (± 5.48)	1.50 (± 0.31)	1.09 (± 0.45)
Periapical radiolucency	8.84 (± 1.52)	26.16 (± 2.49)	0.76 (± 0.13)	0.82 (± 0.17)
Horizontal root fracture	6.60 (± 0.82)	22.93 (± 1.84)	1.16 (± 0.12)	0.79 (± 0.14)
Uncomplicated crown fracture	20.82 (± 2.21)	38.58 (± 6.37)	0.83 (± 0.32)	1.14 (± 0.55)
p-value	<0.001*	<0.001*	<0.001*	0.251

## Discussion

The aim of this cross-sectional observational research was to employ eye-tracking technology to explore the visual behavior of postgraduate endodontic residents while analyzing CBCT images of TDIs in adolescent teeth. The study revealed that there are variations in the visual metrics of CBCT images depicting different TDIs. In the current investigation, postgraduate endodontic residents displayed a significantly prolonged focus and dwell time within the AOI on CBCT images associated with lateral and extrusive luxation injuries. This finding may be explained by the distinct diagnostic challenges associated with luxation injuries, which necessitate close inspection of teeth-supporting structures. This requires a thorough examination of the three CBCT views “axial, coronal, and sagittal” to detect the existence and location of periodontal widening as well as the presence of concomitant alveolar fracture. Conversely, cases featuring uncomplicated crown fractures were characterized by the shortest dwell times, potentially due to the ease with which these fractures can usually be identified from one CBCT view, mostly coronal images, obviating the need for prolonged examination of the other AOIs in the images.

Building on Brocklebank's proposal of a systematic assessment of medical radiographs, which emphasized thorough coverage and subsequent re-examination of potential areas of abnormality [25], we examined the number of visits to the AOI and found that, although not statistically significant, cases involving lateral and extrusive luxation injuries received a greater number of revisits than other cases. This observation aligns with Brocklebank's method, reinforcing the value of revisiting areas associated with diagnostically challenging conditions. Regarding the entry time into the AOI, the study revealed that patients with uncomplicated crown fractures exhibited the longest mean entry time, approximately 21 seconds. This suggests that residents may have initially focused their attention on the apical region of the tooth before progressing to the crown. This sequential pattern of visual attention aligns with prior research involving periapical radiographs, where 51.8% of participants first fixated on the periapical area, whereas 48.2% of participants fixated on the coronal area [22].

While a substantial body of eye-tracking research has focused on panoramic and periapical radiographs [18-24], studies specifically targeting CT imaging are scarce [25-26]. Koji et al. examined the eye movement of 10 dentists by analyzing CT images under different pathological conditions [26]. Crepps, however, was the first to carry out the only study in the field of endodontics, using a sample of 12 participants who underwent CBCT scans. In his study, participants' gaze behaviors were rigorously recorded, and a detailed qualitative analysis of gaze patterns, along with measurements of scanning parameters, was conducted [25]. The study distinguished three unique strategies for interpreting CBCT scans: "non-systematic/nontargeted," "scanning," and "drilling." Accordingly, he advocated for the adoption of a systematic approach, contending that it improves both the accuracy and efficiency of radiographic interpretation [25]. In the current study, residents were not constrained by a specific time limit for scanning the radiographs. This design was chosen to accommodate slower observers and prevent incomplete scanning, consistent with prior research indicating that imposing time constraints could lead participants to inappropriately increase their scanning speed [22].

Previous studies have explored the impact of patient clinical history on radiographic interpretation [27,28]. They demonstrated that a detailed clinical history indicative of particular diagnoses can result in extended gaze dwell times and elevated reporting of specific radiographic abnormalities. Conversely, the absence of such a history was found to negatively impact the reporting of certain findings. For this reason, in our study, participants were provided with a brief dental history and detailed clinical findings before the radiographic images were obtained. Moreover, this approach aligns with guidelines from the American Association of Endodontists and the American Academy of Oral and Maxillofacial Radiology Joint Position Statement that emphasize the importance of periapical radiographs and clinical examination as a standard of care for the initial evaluation of TDIs [9].

Limitations of the study include that participants were provided with fixed reconstruction images for each case. Although this method does not simulate real practice, it was primarily utilized due to the inherent limitations of our eye-tracking device, namely, its inability to track gaze movements during dynamic volume scrolling. Moreover, it is possible that participants' visual behavior was influenced by their knowledge of dental history and clinical findings. However, participants were provided with clinical information to simulate real clinical practice.

Although the study included endodontic residents from different years of their postgraduate training, this diverse selection allowed us to maintain diversity in experience while ensuring that all participants had undergone comprehensive training on CBCT interpretation and the clinical and theoretical aspects of traumatic dental injuries, with the common factor being that all residents had completed a minimum of nine months in their residency. Moreover, as part of their training, all residents participated in standardized training before analyzing the clinical CBCT cases. This training focused on identifying anatomical landmarks of hard tissues in CBCT, thereby minimizing variability and ensuring that the differences observed in the study were reflective of the participants’ diagnostic skills rather than their familiarity with CBCT technology or anatomy. Such measures contributed to the consistency and reliability of our findings. However, the small number of participants may not adequately account for variability across different levels of expertise among postgraduate endodontic residents.

Future research with a larger sample size should take this into account. Further investigation might explore the variations in visual behavior between specialists (endodontists), and residents might find it useful to leverage this technology further in improving diagnostic skills among trainees. Furthermore, cognitive interpretations were not assessed, as this study was designed to investigate visual behavior rather than diagnostic accuracy. The correlation between visual behaviors and diagnostic accuracy would also be valuable for future research.

## Conclusions

This study revealed significant diversity in visual metrics based on the type of TDI. This implies that certain types of TDIs might necessitate more focused attention to ensure an accurate diagnosis. Endodontic residents might benefit from added education and training on the interpretation of CBCT images of TDIs, especially when luxation injuries are suspected.
